# The Promise of Emergent Nanobiotechnologies for In Vivo Applications and Implications for Safety and Security

**DOI:** 10.1089/hs.2022.0014

**Published:** 2022-10-17

**Authors:** Anne M. Arnold, Ashley M. Bradley, Karen L. Taylor, Zachary C. Kennedy, Kristin M. Omberg

**Affiliations:** Anne M. Arnold, PhD, is a Materials Scientist, National Security Directorate, Pacific Northwest National Laboratory, Richland, WA.; Ashley M. Bradley is a Biomedical Scientist, National Security Directorate, Pacific Northwest National Laboratory, Richland, WA.; Zachary C. Kennedy, PhD, is a Materials Scientist, National Security Directorate, Pacific Northwest National Laboratory, Richland, WA.; Kristin M. Omberg, PhD, is Group Leader, National Security Directorate, Pacific Northwest National Laboratory, Richland, WA.; Karen L. Taylor, MPH, is a Senior Technical Advisor, National Security Directorate, Pacific Northwest National Laboratory, Seattle, WA.

**Keywords:** Dual-use science, Biotech industry, Code of conduct, Biosafety protection, Nanoparticles

## Abstract

Nanotechnology, the multidisciplinary field based on the exploitation of the unique physicochemical properties of nanoparticles (NPs) and nanoscale materials, has opened a new realm of possibilities for biological research and biomedical applications. The development and deployment of mRNA-NP vaccines for COVID-19, for example, may revolutionize vaccines and therapeutics. However, regulatory and ethical frameworks that protect the health and safety of the global community and environment are lagging, particularly for nanotechnology geared toward biological applications (ie, bionanotechnology). In this article, while not comprehensive, we attempt to illustrate the breadth and promise of bionanotechnology developments, and how they may present future safety and security challenges. Specifically, we address current advancements to streamline the development of engineered NPs for in vivo applications and provide discussion on nano–bio interactions, NP in vivo delivery, nanoenhancement of human performance, nanomedicine, and the impacts of NPs on human health and the environment.

## Introduction

The convergence of nanomaterials and technology, inspired by the unique physicochemical properties of nanoparticles (NPs) and materials, has produced the multidisciplinary field of nanotechnology. NPs and nanomaterials range from 1 to 100 nm in at least 1 dimension but may be longer in the other 2.^1,2^ They can be produced naturally (eg, by degradation, weathering, or human activities such as burning fossil fuels) or synthetically; synthetic NPs and nanomaterials are often referred to as “engineered.”^2^

Nanomaterials research and development is driven globally by major players, including China, Europe, Russia, and the United States.^3-6^ In 2000, the United States created a government framework known as the National Nanotechnology Initiative (NNI) to seed the commercialization of nanotechnology.^5,7^ Since its inception, more than US$35 billion has been invested^5,8^ and NNI has served as global inspiration for other nations.^9^ For example, the European Union made a notable investment (US$1.35 billion) in the Graphene Flagship project.^3,10,11^ Likewise, Russia invested US$2.7 billion as of 2018, with a US$190 million net return; its investments are managed by the RUSNANO group,^12^ an entity that “implements state policy for the development of the nanoindustry in Russia, acting as a co-investor in nanotechnology projects, which have substantial economic or social potential.”^13^ The sum of nanotechnology investments in China are less clear,^14,15^ although between 2012 and 2017, China's Strategic Pioneering Program on Nanotechnology reportedly invested more than US$152 million.^14^

The increase in research and development funding has fueled extensive global scientific growth: a search for “nanotechnology” in Google Scholar displays over 1 million publications and patents in the last 2 decades. Nanotechnology publications and patents are being collated into publicly available databases to foster collaboration and increase transparency. Journals such as *Data in Brief*^16^ and *Chemical Data Collections*^17^ have created multiple open access repositories for raw experimental data. In addition, public databases (eg, NBI Knowledgebase,^18,19^ caNanoLab,^18,20^ The Nanodatabase,^21^ and the recently retired Nanomaterial Registry^18,22^) have been launched to share protocols, data, and literature among a diverse audience.^18^

The global community has recognized the need for an approach to nanotechnology regulation to supervise the ethical implementation of NPs and protect human health and the environment.^23^ However, relevant agencies, organizations, councils, and strategies are not globally cooperative and substantial gaps remain within existing national and international policy frameworks^24-26^ (Table 1). Current NP regulations typically use preexisting standards for microscale and macroscale materials. However, these may not be applicable to NPs^57-60^ since the physicochemical properties that make nanomaterials useful also make it difficult to extrapolate long-term effects on human and environmental health.^1^ Additional barriers to NP oversight include disagreements between regulatory committees on the definition of NPs,^61^ nanomaterial diversity and applications,^62,63^ and limitations in mass production of NPs that result in poor quality control.^64^

**Table 1.** Collaborative Global and US Nanoparticle Oversight Frameworks

International				
Organization/Council	Committee/Act/Strategy	Responsibilities/ Goals	Participating Countries	References
Canada–US Regulatory Cooperation Council		Develop consistent policies on NP oversight	Canada, United States	27,28
Organisation for Economic Cooperation and Development	OECD Working Party	Understand properties and risks of NPs	Australia, Austria, Belgium, Canada, Chile, Colombia, Costa Rica, Czech Republic, Denmark, Estonia, Finland, France, Germany, Greece, Hungary, Iceland, Ireland, Israel, Italy, Japan, Republic of Korea, Latvia, Lithuania, Luxembourg, Mexico, Netherlands, New Zealand, Norway, Poland, Portugal, Slovak Republic, Slovenia, Spain, Sweden, Switzerland, Turkey, United Kingdom, United States	27-29
International Organization for Standardization	Technical Committee 229	Establish NP standards	Great Britain, Switzerland^a^	27,30
ASTM International	Committee E56 (Nanotechnology)	Establish NP standards	Canada, India, Italy, United States	31,32
Federal Ministry for Economic Affairs and Climate Action	Bundesanstalt für Materialforschung und -prüfung	Establish NP standards	Germany	33
International Electrotechnical Commission	Technical Committee 113	Standardize nano-based electrotechnical products	Germany, Korea^a^	34,35
Institute of Electrical and Electronics Engineers	Nanotechnology Council	Coordinate and advance nanotechnology	United States^a^	36,37

United States				
Organization	Acts/Strategies	Responsibilities/ Goals	References	
American National Standards Institute Nanotechnology Standards Panel	ANSI-NSP Nanotechnology Standards Database	Establish NP standards	38	
US Environmental Protection Agency	Nanomaterial Research Strategy	Study NPs that pose human and environmental risks	39,40	
	Toxic Substances and Control Act	Review safety of new chemicals	39,41,42	
	Safe Drinking Water Act	Regulate NPs materials in potable water supplies	42,43	
	Federal Insecticide, Fungicide, and Rodenticide Act	Oversee NPs materials used as pesticides	42,43	
	Comprehensive Environmental Response, Compensation, and Liability Act	Provide “Superfunds” to remediate hazardous orphan sites	42,44	
	Resource Conservation and Recovery Act	Control hazardous waste from inception to grave	42,46	
	Clean Water Act	Regulate emissions of materials into surface waters	42,47	
	Clean Air Act	Regulate air emission of materials into air	42,48	
	Nanotechnology Task Force	Determine regulatory approaches for nano-based products	49	
US Food and Drug Administration	Center for Drug Evaluation and Research; Center for Biologics Evaluation and Research; Center for Devices and Radiological Health Federal	Regulate nano-based therapies, products, and devices	28,50-52	
	US National Institute for Occupational Safety and Health Administration	NIOSH Nanotechnology Research Center	Lead the health and safety initiative for nanotechnology	53

^a^ Denotes countries represented by the committee and council members, where some positions are elected or appointed terms (as of August 2022). The International Organization for Standardization and the International Electrotechnical Commission have members in 167^54^ and 88 countries,^55^ respectively, while the Institute of Electrical and Electronics Engineers has chapters in more than 14 countries (as of August 2022).^56^ Note that this is not a comprehensive list of the international or US nanotechnology oversight frameworks. Abbreviations: ANSI, American National Standards Institute; IEC, International Electrotechnical Commission; NIOSH, National Institute for Occupational Safety and Health; NP, nanoparticle; NSP, Nanotechnology Standards Panel.

Due to the complexity of the problem, the overlap of nanotechnology with biological safety and security is easy to overlook. But nanotechnology is enabling new areas with the potential to directly impact human health, such as platform-based therapeutics and human performance enhancement, which makes it imperative that we consider these gaps in our understanding and regulations. In many ways, the issues associated with the increasing use of nanotechnology are similar to those raised by the increasing use of synthetic biology.^65,66^ Due to this, and the overlap of safety and security issues, the health security community should be aware of and proactive in addressing these concerns.

The following review is intended to provide a high-level overview of advances in relevant nanobiotechnology (ie, nanotechnology used for biomedical applications) research and development and offer insight into potential hazards that may arise. Given the breadth of current research and development, it is not intended to provide a comprehensive review of nanoscale science but focuses specifically on the promise and concerns associated with NPs for in vivo use, and outlines concerns with respect to biological safety and security. In this review, we refer to NP biological safety (or biosafety) as the risks to human and environmental health associated with unintentional NP exposure^67^; further, we describe NP biological security (or biosecurity) as the risks to human and environmental health caused by the nefarious application of nano-based technologies.^65,68-70^

## Nanomaterials Are Ideally Suited for Biological Applications

NPs have broadly heterogeneous physicochemical properties such as size, shape, charge, porosity, chemical composition, surface morphology, and stability. They are often classified by their material composition with main classes including carbon-based, lipid-based, polymeric, semiconductor, metallic, and ceramic. This breadth of properties, combined with their small size, makes NPs ideally suited for biological applications (Table 2). NPs are significantly smaller than the average eukaryotic cell and can pass through biological barriers such as cell membranes, tissues, and organs. This is beneficial for applications such as bioimaging, gene therapy, and drug delivery.^99^

**Table 2.** Applications of Nanoparticles for In Vivo Use

Nanoparticles	Size (nm)	Applications	References
Carbon-based Fullerenes, nanotubes, graphene, carbon black	0.7-300	Biosensing, imaging and diagnostics, drug and gene delivery, antivirals, antimicrobial treatment, tissue engineering, therapeutics	70-75
Ceramic-based Silica, alumina, hydroxyapatite	<50	Imaging, drug delivery, catalysis, tissue engineering	76-79
Metal Gold, silver, iron, cobalt nanoparticles	1-200	Drug delivery, biosensing/imaging, therapeutics, biomedical enhancement, antivirals, antimicrobial treatments, antifungal therapies	80-84
Semiconductor Quantum dots, cadmium-telluride, indium phosphide	2-50	Imaging, biosensors	85
Polymeric Chitosan, dendrimers	<15	Imaging and diagnostics, biosensing, therapeutics, drug delivery, tissue engineering, antimicrobial treatments	86-91
Lipid Micelles, liposomes	10-500	Drug and gene delivery, imaging	92-96
Janus^a^	0.7-500	Drug and gene delivery, bioimaging and sensing, tissue engineering	97,98

^a^ Signifies that Janus particles can be a combination of any chemical compositions listed above.

## Nanoparticles Have Tunable Physicochemical Properties

NP properties can be manipulated and tuned for a desired function, such as the ability to carry and release a therapeutic chemical payload, fluoresce at a particular wavelength, or cross the blood–brain barrier, by utilizing typical design processes.^100-102^ The development of synthetic nanomaterials, especially those intended for in vivo use, is often lengthy^103^; however, recent advances may reduce the timescale via sophisticated production and screening techniques. For instance, an autonomous platform that leverages Darwinian evolution has demonstrated great utility in the synthesis of gold NPs with programmable shapes. The platform uses a robotic component to synthesize NPs, with spectroscopic analysis to ascertain shape. The spectroscopic analysis uses a genetic algorithm that makes autonomous decisions for the optimization of synthetic conditions to generate shapes of interest. The platform proceeds through cycles of evolution until the desired NP shape is achieved.^104^

High-throughput screening using modeling,^18^ dynamic evolution,^105^ and libraries^103,106,107^ are also being investigated. In 2019, researchers at Northwestern University and the Air Force Research Laboratory reported their method to screen megalibraries of millions of NPs with distinct composition and size.^103^ Gold and silver NPs were formulated into inks that were deposited onto a substrate array using a spray lithography technique. The resulting arrays were used as nanoreactors to catalyze the growth of carbon nanotubes. The catalytic activity of the nanoreactors was screened in a high-throughput fashion using Raman spectroscopy, enabling researchers to identify NPs with optimal catalytic activity based on the composition, size, and spatial distribution of NPs.^103^ High-throughput screening methods have been investigated for optimizing lipid or polymer nanoparticles for therapeutic delivery of proteins, oligonucleotides, small interfering RNA (siRNA), and messenger RNA (mRNA). These methods will ultimately reduce the time required to develop NP-stabilized therapeutics, such as mRNA and protein vaccines.

## Nanoparticle Properties Dictate In Vivo Lifecycle

**Figure 1.**
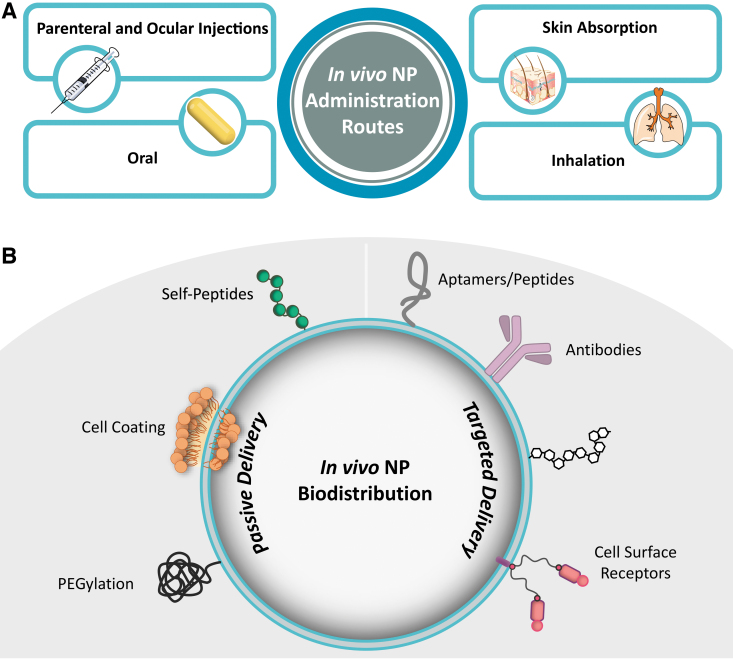
Overview of in vivo (A) administration routes of nanoparticles and (B) biodistribution strategies of nanoparticles (ie, passive and targeted delivery) using coatings. Note that passive delivery strategies demonstrated here rely on stealth coatings to bypass in vivo clearance mechanisms to increase circulation time. Abbreviation: NP, nanoparticle.

The physicochemical properties of NPs govern in vivo delivery, biodistribution, metabolism, and clearance, and dictate possible therapeutic applications.^108^ There are numerous reviews that evaluate how the physicochemical properties of nanomaterials influence nano–bio interactions.^108-110^ Delivery routes into the body for NPs are comparable to traditional routes: parenteral and ocular injections, skin absorption, inhalation, and oral delivery (Figure 1A).^108,111-113^ NP distribution within the body, or biodistribution, can be accomplished using passive or active delivery. Passive biodistribution relies on undirected (passive) delivery to the target.^108,114^ It can be enhanced by cloaking the material (eg, coating NPs with polyethylene glycol, also known as PEG)^115^ to prevent clearance from the body. Active biodistribution methods use targeting mechanisms (eg, carbohydrates or antibodies) to preferentially direct NPs to specific sites (Figure 1).^114,116^

Metabolism and clearance of NPs is not completely understood, but is known to be facilitated by the kidneys, liver, mucosa, and so on.^108,113^ The ability of NPs to stabilize molecules is attractive for therapeutic delivery and in vivo applications; however, some NPs appear to persist indefinitely through encapsulation in tissues.^113,117,118^ As in vivo monitoring methods evolve, a better understanding of metabolism and clearance will be essential to enable efficient targeted distribution, and to ensure biosafety and biosecurity.

## In Vivo Nanotechnology Holds Promise and Potential Concerns

Adoption of nano-based products for in vivo use has been slowed by the difficulty of assuring long-term safety. To date, long-term compatibility studies on the metabolism of NPs are sparse, stemming from issues with NP production and detection.^108,119^ Precise control of NP production is a challenge, resulting in NPs with heterogenous physicochemical properties.^108^ The range of properties complicates biocompatibility studies because different combinations of NPs in a payload could produce vastly different behavior in vivo. Further, traditional methods to study the metabolism of drugs are not sufficient to analyze the metabolic cycle of NPs. Carbon-based NPs, although attractive for in vivo applications, are arduous to differentiate from the carbon-rich environment of the surrounding tissue, complicating the study of biodistribution, metabolism, and clearance. In addition, nanomaterials often persist much longer than in vitro cell culture experiments and the lifetimes of model organisms, so full clearance from the model system may not be observed.^119^

### Nanoparticle Technology to Advance Treatment of Human Disease

Nanomaterial development for in vivo applications has already generated sophisticated technology, such as NP-stabilized mRNA and protein vaccines for COVID-19. NPs have also been applied in research settings to facilitate genomic editing, alter drug potency, and manipulate the immune response.^1,2^ Here, we intend to highlight some interesting current and future nano-based technologies for nanomedicine, but this discussion is not intended to provide a comprehensive list of technologies, which are covered in other reviews.

#### Nanoparticles Can Enhance the Precision of Genomic Editing

Genomic editing offers the promise of a permanent solution to disease or disability as an alternative to surgery or medication. However, some clinical applications have been hindered by inefficient in vivo delivery of the gene editing machinery. Gene editing machinery, such as Clustered Regularly Interspaced Palindromic Repeats (CRISPR), consists of a large protein and nucleic acid component, neither of which readily cross cell membranes.^120-123^ Because NPs readily cross cell membranes, researchers have begun to use NPs to deliver gene editing constituents with greater efficacy.

Several comprehensive reviews detail the state of the art of NP-mediated gene editing.^123-126^ To briefly summarize, lipid-, polymeric-, and gold-based NPs are the most widely studied in vivo CRISPR delivery systems. Targeted delivery strategies have proven to be effective, and in some cases, have been designed so that stimuli (eg, magnetic fields) trigger the release of the CRISPR payload.^124^

NP-CRISPR systems have been designed to target specific cell-types, tissues, and organs in animal models.^127^ Strategies using NP-CRISPR systems have been developed to understand disease and improve treatment methods for genetic disorders,^128^ certain cancers,^128,129^ and other conditions. An interesting example of recent achievements in the NP-mediated delivery of CRISPR is a promising intrauterine gene editing method to treat mice that model human β-thalassemia (a blood disorder). The study demonstrated that gene editing using poly(lactic-co-glycolic acid) NPs encapsulating therapeutic payloads could treat disease even before birth.^127^

#### Nanoparticles Can Enhance or Decrease Drug Potency

Improving drug delivery systems via NPs is a major area of research. Multitudes of studies have demonstrated the use of NPs for superior, targeted delivery of therapeutics to enhance drug potency; the diversity of NPs used for therapeutic delivery is too extensive to summarize in a review, but include NP classes such as carbon-based, lipid-based, polymeric, and ceramic.^128-135^

One of the more interesting areas of nanobiotechnology drug delivery may be the use of NPs that can cross the blood–brain barrier, an inherently difficult endeavor. Most research in this area has focused on using polymeric or magnetic NPs to translocate therapeutics into the brain^136-142^ for greater efficacy in treating neurodegenerative diseases.^140-142^ The ability of NPs to cross the blood–brain barrier could revolutionize brain imaging and treatment for diseases like Alzheimer's disease and glioblastoma. However, the development of NPs to circumvent a relatively impermeable biological barrier raises significant peripheral concerns.

#### Nanoparticles Can Be Used to Modulate Immune Response

NPs can be engineered to avoid recognition from the immune system or to directly influence an immune response.^143,144^ For example, researchers have identified a way to slow the response of macrophages to prevent rapid clearance of foreign, polystyrene nanobeads.^145^ Tagging the NPs with peptides recognized by phagocytes as “self” allowed the NPs to evade immune system and exhibit greater persistence.

The NVX-CoV2373 vaccine pioneered by Novavax for COVID-19 was developed using a proprietary NP-mediated delivery system known as Matrix-M to enhance the immune response.^146,147^ These types of immune system modulation could ultimately be used to enhance drug delivery efficiency and imaging.

#### “Switchable” Nanoparticles

Numerous researchers have been investigating possibilities for “switching” the activity of an NP on or off in vivo. Methods that induce an NP to switch behavior between active and inactive states have been developed using intrinsic or extrinsic stimuli. Intrinsic switching methods include changes in internal homeostasis such as variations in pH, osmolarity, permeability, and enzymatic activity.^148^ Extrinsic switching can be achieved by thermal regulation, ultraviolet radiation, ultrasounds, or proximity to a magnetic source.^149^ Switching is an attractive feature for therapeutics but raises concerns, including unintended switching or malicious switch “hacking.”

### Nanoparticle-Enabled Enhancement of Human Performance

Due to their tunable properties, NPs have been used to enhance chemical reactions and physical properties of materials in laboratory settings for decades. Their ability to cross biological membranes makes them equally promising for human performance enhancement. Researchers have been using NPs to enhance human senses and initiate cellular actions with great success. In an earlier section, we also discussed advances in “switching” nanoparticle activity, which may ultimately enable temporary or reversible enhancement.

#### Nanoparticles Can Facilitate Physiological Enhancements

**Figure 2.**
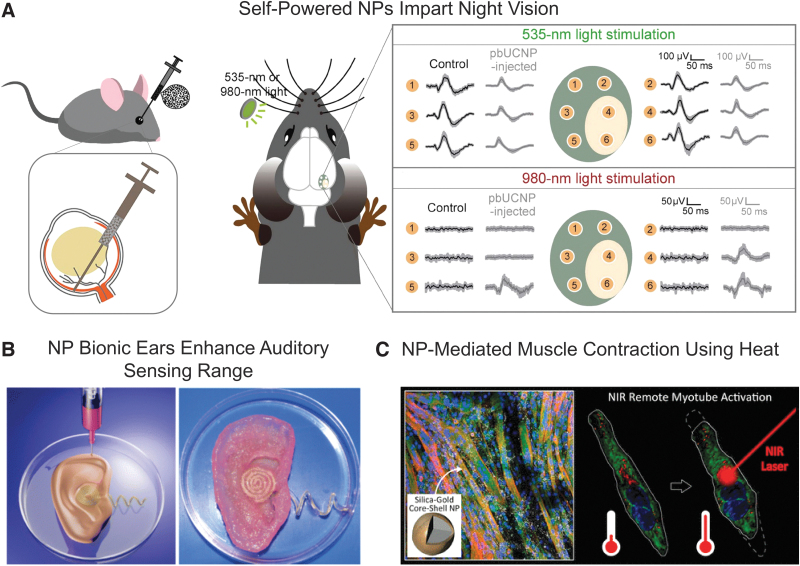
Nanomaterial studies that have enhanced physiological performance. (A) An engineered nanoparticle (pbUCNP) that imparts night vision in mice. Representation of pbUCNP injection into the eye, where it binds with photoreceptor cells in the retina. pbUCNP serves as a self-powered antenna that can be stimulated at 535 nm (day vision) and 980 nm (night vision). Reprinted with permission from Ma Y.^150^ (B) A 3D printed bionic ear composed of a hydrogel laden with cells and conductive nanoparticles coupled to electrodes for auditory sensing. The bionic ear demonstrated enhanced auditory sensing when compared with human hearing. Reprinted with permission from the American Chemical Society.^152^ (C) In vitro study with muscle cells doped with gold nanoshells (ie, NPs consisting of silica coated with a thin layer of gold), where the NPs induced muscle contraction with an externally applied heat source. Reprinted with permission from the American Chemical Society.^156^ Abbreviations: NP, nanoparticle; pbUCNP, photoreceptor-binding upconversion nanoparticle.

Enhancing or repairing damaged human senses (ie, smell, taste, touch, sight, hearing) is an active area of research. Multiple groups are investigating the use of NPs to enhance or repair vision. One set of researchers successfully imparted “night vision” to mice by injecting engineered NPs into the eye, where they bound to the photoreceptor cells in the retina. In vivo, the bound NPs acted as self-powered antennae that converted infrared light into perceptible vision with no impact upon day vision (Figure 2A).^150^ In 2018, a team at Bar-Ilan University reported nanomaterial-mediated vision repair: drops directly applied to the eye that repair near- and farsighted vision by altering the corneal refractive index, creating an alternative, possibly permanent, method to replace glasses, contact lenses, or surgery.^151^

NPs are also being used to enhance hearing. A collaborative group at 2 academic universities developed a bionic ear with superior auditory sensing (Figure 2B).^152^ The researchers 3D-printed the ear using a cell- and conductive NP-laden hydrogel integrated with electrodes. The ear was able to detect radio frequencies well outside the normal range of human hearing. The team was also able to create complementary ears (right and left) that cooperated to listen to audible music. The bionic ears, while a proof-of-concept demonstration, could be used in the future for organ replacement or to enhance the range of auditory communication.

Physiological enhancements using NPs are not limited to the senses. NPs have been used to promote muscle recovery in in vivo animal models^153-155^ and enhance existing muscle function in vitro.^156^ Muscle recovery studies in mice have typically focused on using NPs as carriers and delivery systems for therapeutics that induce muscle repair such as mRNA,^153^ cytokines,^154^ and growth factors.^155^ However, a team of researchers used an in vitro muscle cell line to enhance muscle function using the intrinsic properties of gold nanoshells (NPs with a silica core coated in a thin layer of gold). The team showed that exposing nanoshell-doped muscle cells to near-infrared light (ie, heat) within physiological temperature ranges induced muscle contraction (Figure 2C). This wireless stimulation of muscle cells used a unique mechanism distinct from natural muscle contraction.^156^

#### Nanoparticles May Be Developed to Facilitate Cognitive Enhancements

The development and implementation of NPs that enhance cognitive function has yet to be realized. However, recent advances on the micro- and macro-level with neural–machine interfacing provide the building blocks necessary to develop this technology on the nanoscale. A noninvasive brain–computer interface to control a robotic arm was developed by teams at 2 universities.^157^ A US-based company, Neuralink, is at the forefront of implementing implantable, intracortical microelectrodes that provide an interface between the human brain and technology.^158,159^ Utilization of intracortical microelectrodes may ultimately provide thought-initiated access and control of computers and mobile devices, and possibly expand cognitive function by accessing underutilized areas of the brain.^158^

### Nanobiotechnology Raises Biosafety and Biosecurity, Ethical, and Environmental Quandaries

Nanobiotechnology is enabling advances in genome editing and therapeutics stabilization and delivery and may one day enable modulation of the immune response and human performance enhancement. These are enormous scientific accomplishments; however, these technologies generate a litany of biological safety and security concerns as well as ethical issues. The scientific community is still grappling with the biosafety, biosecurity, ethics, and legality of CRISPR-enabled performance enhancement and “biohacking.” For example, many nations are apprehensive of human genome editing and ban its practice^160,161^; yet in 2018, a Chinese scientist announced the birth of 2 babies whose genomes were edited to be more resistant to HIV infection.^161-163^ In response, the World Health Organization called for development of an international governance framework for human genome editing.^164^ NP-enabled human biohacking brings a new aspect to this type of problem. Considering the breadth and complexity of the ethical concerns regarding in vivo use of NPs, we refer readers to the *NanoEthics* journal,^165^ which provides a multidisciplinary platform to discuss the ethical and social implications of NP technologies.

#### Nanobiotechnology Raises Biological Safety and Security Concerns

**Figure 3.**
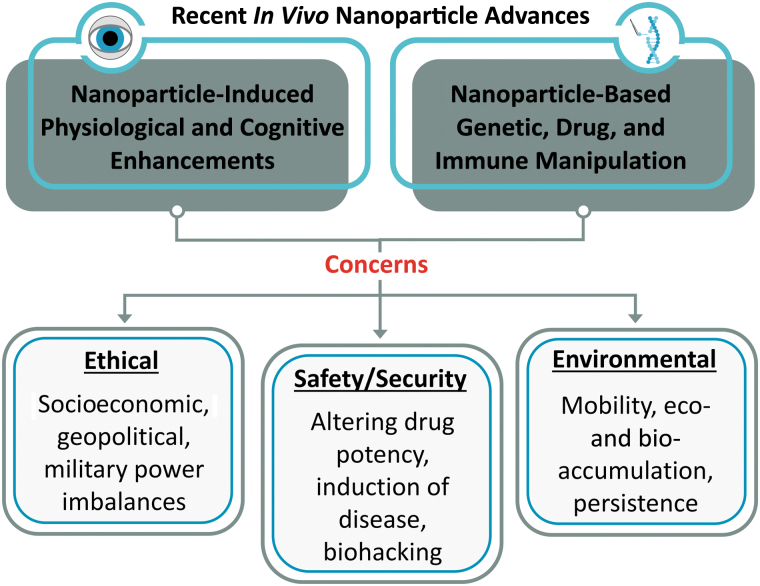
Ethical, safety and security, and environmental concerns arising from NP-mediated physiological or cognitive enhancements and NP-mediated manipulation of genetics, drugs, and the immune system. Abbreviation: NP, nanoparticle.

NPs have intrinsic properties that enable them to pass through biological barriers including cell membranes, organs, and the blood–brain barrier, and many researchers are working to enhance and direct these properties. While this work will enable advanced biomedical applications, it significantly increases biosafety concerns (Figure 3). Researchers handling NP-enhanced therapeutics should consider short-term and long-term effects of exposure to both the individual and combined materials. The mechanisms by which NPs are removed from the body are not completely understood; but, because NPs are often picked up by phagocytic cells, they have been postulated to produce unintended effects such as immunostimulation or immunosuppression, which could result in allergic reactions, chronic inflammation, and potential disease.^166^

NPs that facilitate delivery of therapeutics across the blood–brain barrier also present unique biosafety security concerns. The molecular pathways that dictate cognition and memory formation are not completely understood, but research has implicated that small molecules (eg, formaldehyde) influence these pathways.^167,168^ Human health could be negatively impacted by unintentional or nefarious exposure to chemicals that inhibit memory^167^ or sequester chemicals needed for memory formation.^168,169^ NPs may also increase the permeability of the blood–brain barrier, which is associated with neurological disorders.

The ability to manipulate drug potency with NPs raises biological safety and security concerns. Drug delivery methods developed for therapeutics, such as pain relief, ^170-172^ ultimately could be adapted to trigger side effects or increase potency of commonly abused substances such as narcotics and opioids. With the drug epidemic at an all-time high, NP technology could be used to increase addiction numbers and the severity of a user's dependency, resulting in more overdose-related deaths. NPs could also be used to alter over-the-counter products to increase potency and the possibility of side effects.

Novel technologies are vulnerable to ethical asymmetries and unintended use, creating concerns that nanobiotechnology could be used to cause deliberate damage to human health. The capability to hack human health-related technology has already been demonstrated on the macro- and microscale. “White hacker” proofs by government agencies and academic institutions of medical devices such as magnetic resonance imaging (MRI) machines^173^ and implantables (eg, pacemakers, insulin pumps,^173^ and neurostimulators)^174^ enabled with wireless technology have created disquiet in the medical and security communities. Deliberate “hacking” of NPs that can be switched by thermal regulation, ultraviolet radiation, ultrasounds, or proximity to a magnetic source could be used to activate or deactivate a critical medical function. These types of biosecurity risks will continue to increase as NP-enabled medical technologies come to market.

#### Nanobiotechnology Raises Ethical Concerns

Numerous researchers have raised ethical issues associated with in vivo nanobiotechnology. In 2019, a special edition of the *AMA Journal of Ethics* explored a variety of issues associated with nanomedicine, ranging from helping patients understand the unknowns associated with NP-enabled medicines to identifying violations of individual privacy (eg, use of nanomedicine to track prescription drug compliance).^175^ More recently, the concentrated distribution of NP-stabilized COVID-19 vaccines in wealthy countries has been questioned.^176^ Like any advanced technology, nanobiotechnology has the potential to exacerbate socioeconomic imbalances. If used nefariously or to enhance human performance, it might also create significant geopolitical or military power imbalances (Figure 3).

#### Nanobiotechnology Raises Environmental Concerns

NPs are common constituents in cosmetic, electronic,^177^ optic, automotive,^178^ wound dressing,^176,179^ surgical equipment,^179^ and food products.^180^ As a result, they are commonly distributed throughout the environment. The extensive use of NPs in consumer goods raises concerns about mobility, accumulation, and persistence of nanomaterials in the environment (Figure 3).^177^ These concerns will be exacerbated by the use of NPs in in vivo applications.

Sunscreen has become a major source of unintentional NP pollution. Sunscreen contains titanium dioxide (TiO_2_) and zinc oxide (ZnO) NPs that reflect, scatter, and/or absorb ultraviolet rays.^181^ TiO_2_ and ZnO are considered safe for topical use^182^ because they are not soluble and do not absorb through the skin. However, the increasing use of NPs in sunscreens has resulted in the distribution and accumulation of TiO_2_ and ZnO in water and soils.^183^ While TiO_2_ and ZnO are considered safe for topical usage, chronic exposure to animals through inhalation and ingestion causes an onset of health issues that can lead to aggregation into tissues.^184,185^ TiO_2_ particles have been shown to cause oxidative stress that damages brain cells in model organisms.

In part due to their ubiquity and ease of production, ZnO and TiO_2_ NPs also are being considered for in vivo applications. There is great interest in using TiO_2_ as a photosensitizer for photodynamic therapy for diseases ranging from cancer to psoriasis.^186^ ZnO is being considered as an antitumor therapeutic, although the mechanism of toxicity in tumor cells is not well understood.^187^ While in vivo applications of these NPs are unlikely to drive pollution compared with sunscreen, these uses highlight both growing interest and the uncertainties associated with environmental accumulation of biologically active NPs.

Silver NPs have a long history of use for their biological activity, specifically their antimicrobial properties, and are in widespread use in products^185,186^ such as water filters,^185^ cosmetics,^188,189^ toothpaste,^188^ wound dressings, and surgical instruments.^189^ To date, silver NP safety has not been properly established,^188^ although an increasing number of studies have emphasized their toxicity^189^ and associated silver NP exposure with health risks^188-191^ such as encapsulation in lung tissue,^189^ oxidative stress, DNA damage,^191^ inflammation,^189^ and cognitive impairment.^188^

The increasing risk of engineered nanomaterial accumulation in the environment and unintended exposure has galvanized policymakers to begin the inception of regulatory NP policies. France banned the use of TiO_2_ in food products beginning in January 2020.^192^ In October 2021, the European Commission amended certification of certain TiO_2_ powders as a Category 2 suspected carcinogen.^193^ The Canadian General Standards Board implemented a more comprehensive approach to NP regulations, banning all NPs from the production and preparation processes of organic food.^194^ These policies do not address other families of nanomaterials or other consumer products.^59^

Ultimately, NP environmental pollution or deliberate contamination are also biosecurity concerns. Because NPs can aggregate in water and sediment, aquatic organisms used as food sources may accumulate contaminants, creating short- and long-term deleterious effects on the food chain and ecosystem. Accidental or deliberate environmental dispersal of NPs could also render areas unsafe for agricultural use.

## Conclusion

The convergence of nanomaterials, technology, and biology holds tremendous promise. NPs enable superior strategies compared with traditional microscale materials for many biomedical applications, including gene therapy, drug delivery, and bioimaging/biosensing. Many of the same properties that make nanomaterials excellent candidates for in vivo use also raise biological safety and security concerns and could be intentionally exploited for harmful activities. Because of this potential for harm, awareness of concerns and threats arising from nano-based research is becoming increasingly important.

The recent widespread use of NP-stabilized vaccines for COVID-19 is just one example of the utility of nanobiotechnology to transform human health. However, the long-term biological safety and security effects of these technologies should be an active area of consideration, specifically in the realm of policy regulation. Unfortunately, regulation of nano-based technologies is not sufficient. The policy framework is often segmented by locale (eg, policies in the United States differ greatly from those in European countries), and within these segmented frameworks, there is a lack of interagency overlap to address the regulation of NPs. For example, in the United States, nanomedicines are regulated by the US Food and Drug Administration, but the persistence of these therapies and their effects on the environment is often not considered or regulated by the US Environmental Protection Agency. Thus, we suggest the development of a more cooperative, global policy framework that considers the heterogeneity and persistence of NP-based technologies.

The development of a global policy framework for nanobiotechnology will not be an easy feat and requires a proactive approach to continually identify short- and long-term biosafety and biosecurity risks associated with NP usage. The synthetic biology community has developed a unique approach to identify biological safety and security issues associated with new technologies using the International Genetically Engineered Machine (iGEM) competition. iGEM is set up broadly to cover synthetic biology as a whole and is governed by an experienced panel of judges and coaches and defined rules. Medical- and pharmaceutical-focused nanobiotechnology is a smaller field than what iGEM encompasses, and therefore adoption of a similar approach would need to be scaled and focused to be most effective.
